# Non-invasive index of liver fibrosis induced by alcohol, thioacetamide and schistosomal infection in mice

**DOI:** 10.1186/1471-230X-10-53

**Published:** 2010-06-01

**Authors:** Mohamed H Hessien, Ismaiel M El-Sharkawi, Ahmed A El-Barbary, Doha M El-Beltagy, Ned Snyder

**Affiliations:** 1 Department of Chemistry, Faculty of Science, Tanta University, Tanta 31111, Egypt; 2 Department of Zoology, Faculty of Science, Tanta University, Tanta 31111, Egypt; 3 Division of Gastroenterology at the University of Texas Medical Branch (UTMB) in Galveston, TX: 77555, USA

## Abstract

**Background:**

Non invasive approaches will likely be increasing utilized to assess liver fibrosis. This work provides a new non invasive index to predict liver fibrosis induced in mice.

**Methods:**

Fibrosis was generated by thioacetamide (TAA), chronic intake of ethanol, or infection with *S. mansoni *in 240 mice. Both progression and regression of fibrosis (after treatment with silymarin and/or praziquantel) were monitored. The following methods were employed: (i) The METAVIR system was utilized to grade and stage liver inflammation and fibosis; (ii) Determination of hepatic hydroxyproline and collagen; and (iii) Derivation of a new hepatic fibrosis index from the induced changes, and its prospective validation in a group of 70 mice.

**Results:**

The index is composed of 4 serum variable including total proteins, γ-GT, bilirubin and reduced glutathione (GSH), measured in diseased, treated and normal mice. These parameters were highly correlated with both the histological stage and the grade. They were combined in a logarithmic formula, which non-invasively scores the severity of liver fibrosis through a range (0 to 2), starting with healthy liver (corresponding to stage 0) to advanced fibrosis (corresponding stage 3).Receiver operating characteristic curves (ROC) for the accuracy of the index to predict the histological stages demonstrated that the areas under the curve (AUC) were 0.954, 0.979 and 0.99 for index values corresponding to histological stages 1, 2 and 3, respectively. Also, the index was correlated with stage and grade, (0.947 and 0.859, respectively). The cut off values that cover the range between stages 0-1, 1-2 and 2-3 are 0.4, 1.12 and 1.79, respectively. The results in the validation group confirmed the accuracy of the test. The AUROC was 0.869 and there was good correlation with the stage of fibrosis and grade of inflammation.

**Conclusion:**

The index fulfils the basic criteria of non-invasive marker of liver fibrosis since it is liver-specific, easy to implement, reliable, and inexpensive. It proved to be accurate in discriminating precirrhotic stages.

## Background

The end stage complications of liver disease including cirrhosis, and portal hypertension are related to advanced fibrosis [1]. Management decisions in patients with chronic liver diseases often depend upon the stage of liver fibrosis. Hence, the accurate estimation of fibrosis is important for the prevention of the subsequent complications. For 60 years, liver biopsy has been regarded as a gold standard diagnostic method for assessing liver fibrosis. Despite its longstanding utility, liver biopsy has some disadvantages which include its invasive nature [2], expense, as well as inter observer variability. It also has some negative features such as resistance of patients to undergo liver biopsy due to the discomfort [3], possible complications [2], and sampling error due to inadequate liver specimen length or fragmentation [4,5]. Therefore, alternative and accurate non invasive means to estimate fibrosis are needed.

Multiple hepatic fibrosis markers utilizing either simple blood tests or measurements of components of the extracellular matrix (ECM) have been proposed, and some are in clinical use. Imbert-Bismut and colleagues [6] proposed an index combining 5 variables (bilirubin, gamma-glutamyltranspeptidase (γ-GT), haptoglobin, alpha 2-macroglobulin, and apoliprotein A1). The index ranges from 0-1 and currently is marketed as the *FibroTest*. Subsequently, other hepatic fibrosis markers have been proposed such as the Forns index [7], the AST/platelet ratio index (APRI) [8], and the European or Enhanced Liver Fibrosis Index [9]. These tests generally separate mild from significant fibrosis or mild/moderate from advanced fibrosis. Apart from Fibrotest, they usually have 2 cut offs with an indeterminate zone, where the accuracy is less. They can also be combined in decisional algorithms [10,11]. Another non invasive tool is transient elastography, either alone or combined with a hepatic fibrosis marker such as the Fibrotest [11]. Other investigators have suggested for best accuracy combining both a simple index and an ECM index [12,13]. Such approach expands the number of investigations and increases the cost required to stage the degree of fibrosis.

The majority of publications have been concerned with fibrosis among HCV infected patients. Also, few studies have monitored the regression of drug-mediated fibrosis. This triggered our interest to establish a non-invasive index to score the liver fibrosis in mice models induced by injury from a hepatotoxin (thioacetamide, TAA), chronic intake of alcohol or Schistosomal infection. Moreover, we employed the index to monitor the reversibility of fibrosis in mice treated with silymarin and/or praziquantel (PZQ). The index includes four serum biochemical markers (3 liver-specific and one oxidative stress) combined in a logarithmic formula derived from values of both tested and normal control mice.

## Methods

### Animal grouping

The study was carried out on 310 albino mice (MC1 strain), weighing 17.78-26.62 g, They were kept in breeding cages, and they received similar basic care with a standard diet. The test set included 240 mice, whereas the validation set included 70 mice. Humane and ethical animal practices were followed that were under the standard regulations dictated by the animal care committee of School of Science, Tanta University.

#### 1-Test groups

**Figure 1:**
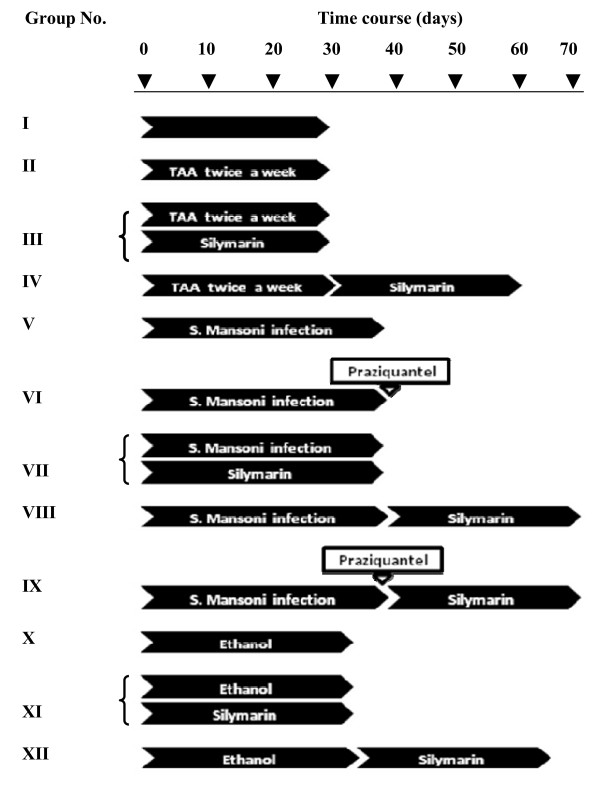
**Time course of exposure of mice to different liver fibrotic inducers and treatments**. Mice were categorized into 11 groups according to the fibrotic inducer with or without treatment, in addition to normal control group

The test group of 240 mice was categorized into 12 groups (20 mice each): Group I included normal control mice. Group II included mice intraperitonealy injected with TAA. In group III mice were simultaneously treated with both TAA and silymarin, whereas in group IV mice were injected with TAA, for a month, then treated with silymarin for a similar period. Group V mice were infected with *S. mansoni*. In group VI, after being infected with *S. mansoni*, mice were treated with PZQ. Group VII mice were treated with silymarin for a month, starting the day after infection with *S. mansoni*. In groups VIII and IX, after being infected with *S. mansoni*, mice were treated with silymarin or PZQ + silymarin, respectively. Group X mice were given ethanol, whereas groups XI and XII they were treated with silymarin during or after ethanol intoxication, respectively. Figure 1 shows the time, course of fibrogenesis, and treatments in different groups.

#### 2-Validation groups

70 mice were used to validate the proposed index (validation group). These animals were categorised into 4 subgroups. Three groups (20 mice each) were treated with TAA, infected with *S. mansoni *or intoxicated with ethanol similar to groups I, V and X, respectively. Animals of these groups were sequentially sacrificed, where 5-7 animals were killed every 10-15 days. The fourth group (10 mice) was untreated and used as a normal control group.

### Induction and treatment of liver fibrosis

TAA-induced liver fibrosis was generated in mice by intraperitoneal injection of 200 mg/Kg TAA (Sigma-Aldrich Chemical Co., USA) twice a week for a month [14]. *S. mansoni*-induced liver fibrosis was generated by immersion of mouse tails into a suspension of 50 cercaria for 30 minutes. Cercaria usually develop into larva, and subsequently into an adult worm in about 42 days [15]. To develop ethanol-induced liver fibrosis, ethanol (10%) was given to the mice in drinking water [16] for at least 35 days. Silymarin (Sedico Co., Egypt) was dissolved in water (140 mg/dl) and taken orally [17]. As a reference antischistosomiasis drug, mice (in groups VI and IX) were orally injected by lavage with a single dose of 600 mg/Kg of mice body weight of PZQ (Biltricide, Epico Co., ARE). In the validation group (*n = 70*), liver fibrosis was similarly induced like test groups, then animals were given code numbers. Both the histological anlaysis and the score value were independently caluculated for each mouse.

### Sampling

At the end of treatment period, animals were sacrificed, where both blood and the liver tissues were collected. Serum was recovered for biochemical investigations and liver homogenate was prepared and used for determination of hepatic variables. In addition, a portion of the liver was preserved in 10% formalin and used in histopathological analysis.

### Biochemical markers

Serum total bilirubin, gamma glutamyltransferase (γ-GT) (E.C.2.3.2.2.) activity, and total proteins (TP) were estimated using the commercially available reagents following the manufacturer instructions. Serum level of reduced glutathione (GSH) was estimated according to Ellman [18]. The method is based on the oxidation of GSH by 5,5'-dithiobis-2-nitrobenzoic acid, [DTNB], and the resultant yellow colored ion, is measured at 412 nm. Liver homogenate (10%) was prepared and used to estimate total malondialdehyde (MDA) according to Okhawa and Ohishi [19]. The method depends on the reaction of MDA with thiobarbituric acid (Sigma-Aldrich Chemical Co., USA). Also, hydroxyproline (HP) content of collagen was determined in liver according to Bergman and Loxlely [20] and Medugorac [21], where HP was hydrolyzed with HCl, oxidized into pyrole and coupled with p-dimethyl-aminobenzaldehyde (Sigma-Aldrich Chemical Co., USA) forming a red color, which is measured at 558 nm. The collagen content was calculated by multiplying the HP concentration by 7.46, where this imino acid represents 13.4% of collagen. Tissue transglutaminase activity was estimated in 100 μl liver homogenate, by a direct spectrophotometric method developed by DeMacedo and co-authors [22]. The method uses N,N-dimethyl-1,4-phenylenediamine (DMPDA) (Sigma-Aldrich Chemical Co., USA) as γ-glutamyl acceptor substrate and carbobenzyloxy L-glutamylglycine (Z-Gln-Gly) (Sigma-Aldrich Chemical Co, USA) as a peptide γ-glutamyl donor substrate. The resulting anilide, substituted with a strong electron-donating group, is a chromophore that absorbs light at 278 nm. Thus, the transamidation activity of transglutaminase could be determined kinetically over 7 minutes period by measuring the increase in absorbance. A routine laboratory method was used in determination of proteins in liver homogenate assayed according to Lowry et al., [23] using bovine serum albumin as a standard.

### Histological and non-invasive scoring of liver fibrosis

In order to analyze the histological features of liver according to the METAVIR scoring system, the standard protocol of tissue staining [24] was applied, where liver tissues were fixed, paraffin-embedded and stained with at least haematoxylin and eosin. Slides were examined by a single pathologist unaware of animal treatments. Every specimen was staged according to F0 to F4 system: F0 = no fibrosis, F1 = portal fibrosis without septa, F2 = few septa, F3 = numerous septa without cirrhosis, and F4 = cirrhosis [25]. Histological activity (a measure of necroinflammatory lesions) was graded as follows: G0 = no histological activity, G1 = mild activity, G2 = moderate activity, and G3 = severe activity.

### Statistical analysis

Both serum and hepatic variables are expressed as a mean (± standard deviation). The normal distribution of initial data (bilirubin, TP, γ-GT and GSH) was tested by the Kolmogorov-Smirnov (KS) normality test. Accordingly, the comparison between means were analyzed by the ANOVA test. *P *values less than 0.05 were considered significant. The correlation between index variables and the histological stages and grades were investigated. Also, the correlations between the non invasive score and the corresponding histological stage and grade were estimated by the nonparametric *Spearman *correlation test. Comparisons between the histological stages of diffrent groups were performed with Wilcoxon signed-rank test. The Graphpad Instat software package (Graphpad, San Diego, CA, USA) was used, however reciever operating curves (ROC) and K mean clustring were performed by SPSS-10.0 (strandard version, SPSS Inc.).

## Results

### Biochemical panel

To validate a non invasive model, we investigated two panels of variables. The first "serum panel" included γ-GT, bilirubin, TP, and GSH. Incorporation of ALT, AST or ALP did not improve the model. The second "hepatic panel" included MDA, HP and collagen. As table 1 shows, the bilirubin has increased in mice treated with TAA, alcohol or infected with *S. mansoni *(0.19 ± 0.017, 0.162 ± 0.017 and 0.127 ± 0.019 mg/dl, respectively) compared to the mean level of normal mice (0.015 ± 0.006 mg/dl). Treatment of animals with silymarin and/or PZQ, led to variable degrees of reduction in bilirubin level. The best improvement was achieved in mice treated with silymarin after cessation of intoxication (groups IV and XII) and in mice dual-treated with silymarin and PZQ. Less improvement has occurred in mice treated with the drug(s) during the fibrogenesis process (gps III, VII and XI). Also after treatment, γ-GT decreased to 10.4 ± 0.8, 9.7 ± 0.6 and 10.2 ± 0.97 U/L in groups IV, IX and XII, respectively, similar to the normal control (9.27 ± 0.8 U/L). The hypoproteineamia seen in mice with fibrosis (3.83 ± 0.1, 4.74 ± 0.22 and 4.31 ± 0.2 g/dl) was significantly improved after treatment. GSH levels revealed the development of oxidative stress in mice with fibrosis (gps II, V and X). Treatments of mice with silymarin and/or PZQ after cessation of the fibrosis inducers restored the normal level of GSH, while 97.2%, 99.2% and 98.8% of the normal GSH level was restored in groups IV, IX and XII, respectively (Table 1).

**Table 1:** Levels of serum markers used to formulate the non-invasive fibrosis index

Fibrotic inducer	Group No. (treatment)	Bili-(mg/dl)	γ GT (U/L)	TP (g/dl)	GSH (mM)
	I (Normal)	0.015 ± 0.006	9.27 ± 0.83	6.10 ± 0.10	1.42 ± 0.04

***TAA***	II (TAA)	0.19 ± 0.017a	28.49 ± 1.32a	3.83 ± 0.17a	0.82 ± 0.02a
	
	III (TAA silymarin)	0.037 ± 0.006ab	13.9 ± 0.82ab	5.67 ± 0.22ab	1.34 ± 0.01ab
	
	IV (TAA then Silymarin)	0.024 ± 0.009b	10.42 ± 0.82b	5.98 ± 0.13b	1.38 ± 0.03b

					

***S. mansoni***	V (*S. mansoni *infection).	0.127 ± 0.019a	22.93 ± 0.52a	4.74 ± 0.22a	1.23 ± 0.02a
	
	VI (PZQ after infection).	0.103 ± 0.019a	22.00 ± 0.63a	4.80 ± 0.23a	1.24 ± 0.03a
	
	VII (Silymarin during infection).	0.054 ± 0.008ab	16.91 ± 1.04ab	5.25 ± 0.18ab	1.33 ± 0.03ab
	
	VIII (Silymarin after infection).	0.065 ± 0.008ab	18.30 ± 0.97ab	5.07 ± 0.11ab	1.31 ± 0.01ab
	
	IX (Silymarin after PZQ).	0.017 ± 0.006b	9.73 ± 0.63b	5.95 ± 0.15b	1.41 ± 0.02b

					

***Ethanol***	X (Ethanol).	0.162 ± 0.017a	26.17 ± 1.32a	4.31 ± 0.20a	0.99 ± 0.03a
	
	XI (Silymarin during ethanol intake).	0.028 ± 0.006ab	11.12 ± 1.04ab	5.85 ± 0.23ab	1.34 ± 0.01ab
	
	XII (Silymarin after ethanol intake).	0.022 ± 0.008b	10.19 ± 0.97b	5.95 ± 0.35b	1.40 ± 0.02b

Bil: bilirubin, γ-GT: gamma glutamyltranseferase, TP: Total protein and GSH: reduced glutathione.

Small letters (a) and (b) indicate a significant change of the corresponding group compared to normal and untreated groups, respectively.

In parallel, the increased hepatic MDA in fibrogenated animals (Table 2) improved after silymarin treatment, particularly in TAA and alcohol-induced fibrosis. In *S. mansoni *infected mice, in contrast, neither silymarin nor PZQ had a significant effect, where they minimally (11% and 5%, respectively) decreased the MDA. The improvement in both liver function and the oxidative stress conditions significantly limited the overproduction of hepatic collagen, which decreased to 122.14 ± 0.7, 118.88 ± 9.76 and 119.21 ± 13.55, more or less similar to that of healthy animals (110.80 ± 9.22). Hepatic MDA was negatively correlated with serum GSH (*r*=-0.93) and both MDA and collagen were highly correlated with the non invasive score (Table 3).

**Table 2:** Liver and oxidative stress markers in liver tissue

Inducer	Group No. (treatment)	HP	Collagen	MDA
	I (Normal)	14.84 ± 1.24	110.80 ± 9.22	0.83 ± 0.02

***TAA***	II (TAA)	42.65 ± 3.65a	318.19 ± 27.2a	2.15 ± 0.12a
	
	III (TAA+ silymarin)	17.33 ± 1.97ab	129.24 ± 14.66ab	0.99 ± 0.07ab
	
	IV (TAA then Silymarin)	16.14 ± 1.43b	122.14 ± 10.70b	0.87 ± 0.04 b

				

***S. mansoni***	V *(S. mansoni *infection).	32.40 ± 2.35a	241.83 ± 17.50a	1.39 ± 0.14a
	
	VI (Praziquantel after infection).	31.72 ± 2.68a	236.62 ± 20.02a	1.32 ± 0.05a
	
	VII (Silymarin during infection).	19.28 ± 1.35ab	143.84 ± 10.08ab	1.09 ± 0.07a
	
	VIII (Silymarin after infection).	21.92 ± 1.54ab	163.51 ± 11.47ab	1.14 ± 0.05a
	
	IX (Silymarin after Prazaquantel).	15.94 ± 1.31b	118.88 ± 9.76b	0.85 ± 0.01b

				

***Ethanol***	X (Ethanol).	37.96 ± 3.20a	283.19 ± 23.88a	1.74 ± 0.06a
	
	XI (Silymarin during ethanol intake).	17.70 ± 1.58ab	132.04 ± 11.79ab	0.95 ± 0.02ab
	
	XII (Silymarin after ethanol intake).	15.98 ± 1.82b	119.21 ± 13.55b	0.87 ± 0.03b

HP: hydroxyprolin, TP: total protein, MDA: malondialdehyde.

Small letters (a) and (b) indicate a significant changes of the corresponding group compared to normal and untreated groups, respectively.

**Table 3:** Correlations between non-invasive score and hepatic markers (MDA and collagen)

	Hepatic biochemical variables	
	Hepatic MDA	Hepatic collagen
**Non invasive score of training set**	*0.939, 0.000*	*0.901, 0.000*

### Modelling of hepatic fibrosis marker

Among the multiple biochemical variables studied in the 240 normal and fibrogenated animals in the test group, the most useful were the bilirubin, total protein, γ-GT, and GSH. This was indicated by the high correlation between these markers and both histological stage and grade (Table 4). Subsequently, a logarithmic empirical formula was constructed:

**Table 4:** Correlations between the histological analysis (stage and grade) and each marker of the index panel

Non invasive serum variables	Histology	
	**Stage**	**Grade**
**Bilirubin**	*0.922*	*0.894*
γ**-GT**	*0.942*	*0.893*
**T.Proteins**	*-0.917*	*-0.856*
**GSH**	*-0.898*	*-0.819*

Where: bil = serum total bilirubin, γ-GT = serum gamma glutamyl transferase, TP = serum total protein and GSH = reduced glutathione. Small letters (*t *and *c*), refer to the "test" and "control" samples, respectively. To calculate the non-invasive score, Bilc, γ-GTc, TPc and GSHc were replaced with the corresponding levels measured in normal mice (0.015 mg/dl, 9.27 U/l, 6.1 g/dl and 1.42 mM), respectively. This formula, derived from test set, was also used to assess fibrosis score of validation set.

### Regression of fibrosis stage and inflammatory grade

**Figure 2:**
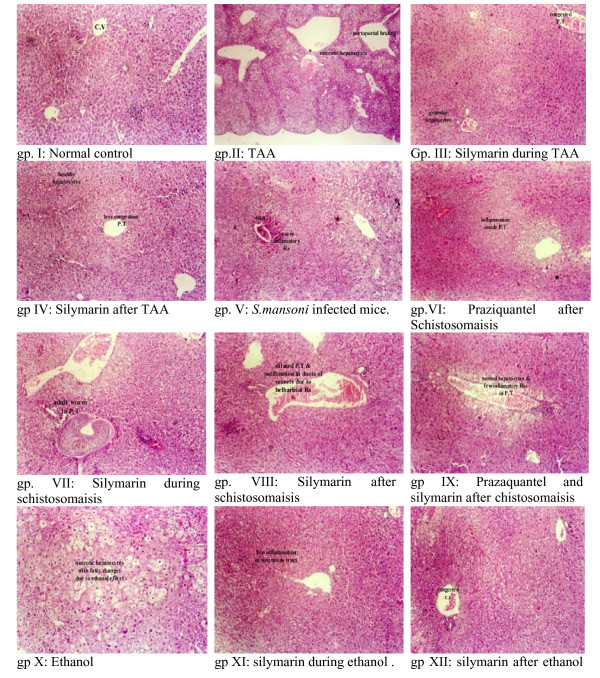
**Histological analysis of mice livers**. TAA-induced liver fibrosis (A) was treated with silymarin during (B) or after (C) TAA. Also, alcohol-induced fibrosis (D) was treated with silymarin during (E) or after (F) alcohol intake. *S. mansoni*-induced fibrosis (G) was treated with praziquatel (H) or praziquantel then silymarin (I) after the development of schistosmiasis

**Figure 3:**
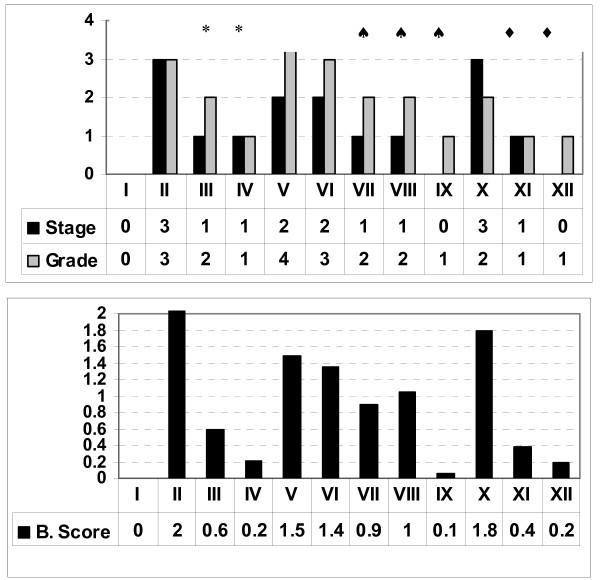
**Regression of fibrosis monitored by histological analysis (Top) and by the non-invasive index (Bottom)**. The degree of fibrosis was evaluated by both the stage and inflammatory grade according to METAVIR system. (*Stars*): refers to significant changes between the corresponding groups versus group II, (*Hearts*) refers to significant changes between the corresponding groups versus group V, and (*Black squires*): refers to significant changes between the corresponding groups versus group X. Also, 4 serum (non-invasive) markers were combined in a logarithmic formula and used to scale the degree of fibrosis in mice of different groups. The scale ranges from 0 (corresponding to normal liver) up to 2 (corresponding to invasive fibrosis). The bottom panel presents the index values of different test groups

In general, the histological analysis of treated animals (Figure 2) revealed the regression of both the stage and the grade of liver histology. Livers of untreated mice had stages 3, 2 and 3. In TAA-induced fibrosis (gp II), the histology revealed portoportal bridging, portal fibrosis, piecemeal necrosis, fatty changes in hepatocytes, portal inflammation and congested central veins. When silymarin was taken during the fibrogenesis stage (gp III), the condition was improved with marked inflammatory reactions in portal tracts, fatty changes in hepatocytes, and no bridging in portal tracts. When the drug was taken after cessation of TAA (gp IV), livers had enlarged hepatocytes without structural changes, less inflammatory reactions in portal tracts and less congestion in central vein. The stage decreased from 3 to 1 (P < 0.05) in both cases. A better regression pattern was observed in the groups treated with alcohol, especially in group XII (gp X *vs *XI or XII, P < 0.05). Slides of *S. mansoni *infected mice (gp V) demonstrated the ova of *S. mansoni *surrounded by severe inflammatory reaction (grade 3) and a congested central vein. Treatment of mice with PZQ (gp VI) slightly reduced the inflammatory reaction to (grade 2), however it did not improve the stage (P > 0.05). More histological improvement was obtained with dual treatment with PZQ and silymarin (gp IX), whereas normal pattern (with normal hepatocytes, few inflammatory reactions in portal tract and no ova or adult worm) was seen in mice treated with both drugs after cessation of *S. mansoni *infection (Figure 3).

### Biochemical scoring of fibrosis

After careful monitoring of the hepatic environment in mice with liver fibrosis (with or without treatment), the serum panel was utilized to formulate the fibrosis index (**Methods**). The average score corresponding to the normal control mice in group 1 (with stage 0) was 0.127 ± 0.027 (range: 0-0.4). Liver specimens with histological stage 1 had scored 0.668 ± 0.16 (ranges from 0.24 to 1.12). Higher average scores were obtained in mice with stages 2 and 3 (1.35 ± 0.05 and 2.03 ± 0.13, respectively). The center of each index range corresponding to each histological stage was determined by K-mean clustering (Table 5), where the mean values corresponding to healthy (F = 0), mild fibrosis (F = 1), moderate fibrosis (F = 2) and severe fibrosis (F = 3) were 0.11, 0.51, 1.11 and 1.85, respectively. Also, the cut off values that cover the range between stages 0-1, 1-2 and 2-3 were 0.4, 1.12 and 1.79, respectively.

**Table 5:** K-mean clustering and cut off values of the index corresponding to different precirrhotic histological stages

Score	Histological stage			
	**0**	**1**	**2**	**3**

**Mean**	0.127 ± 0.03	0.668 ± 0.29	1.350 ± 0.26	2.030 ± 0.13

**K mean clustering**	0.11	0.51	1.11	1.85

**Cut-off**		0.4	1.12	1.79

### Biochemical versus histological scoring

**Figure 4:**
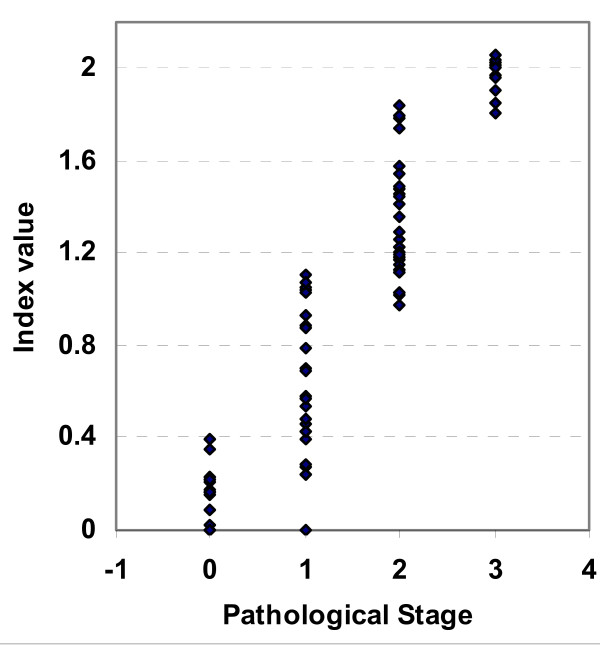
Individual index values corresponding to different histological stages in mice of validation group

The histological analysis of different groups matched the score of the proposed index. Plotting receiver operating characteristic curves (ROC) for index values versus corresponding histological stages (Figure 4) demonstrates that the areas under the ROC curves (AUROC) were 0.954 (95% Confidence Interval 0.889-1.019), 0.979 (95% Confidence Interval: 0.892-1.004) and 0.99 (95% Confidence Interval: 0.0-1.0) for index values corresponding to stages 1, 2 and 3, respectively) (Table 6). Within each category the index was highly correlated with the corresponding stage. In untreated and treated TAA, *S. mansoni *and alcoholism groups the correlation between the index and the histological stages were 0.98, 0.73 and 0.99, respectively.

**Table 6:** Area under receiver operating characteristic curves and range of confidence intervals for both test and validation sets

Animals	Histological Stage	AUROC	Asymptotic 95% Confidence Interval	
			**Lower Bound**	**Upper Bound**

**Training set**	1	0.954	0.889	1.019
	
	2	0.979	0.892	1.004
	
	3	0.989	0.900	1.001

				

**Validation set**		0.869	0.790	0.949

### Validation group

**Figure 5:**
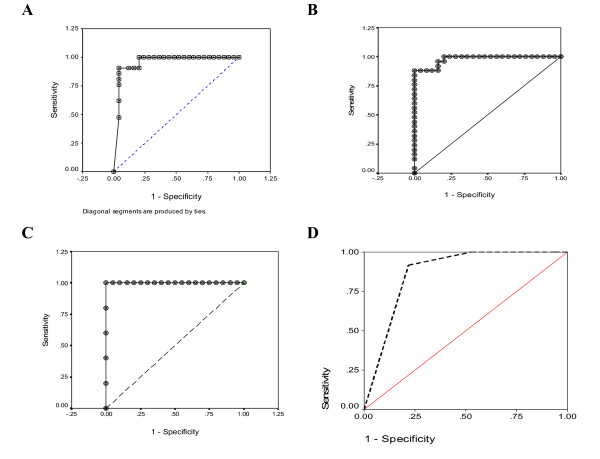
**Receiver operating curves (ROC) of the indices values corresponding to different histological stages**. A, B and C represent the index values covering the range of stage 1 stage 2, and stage 3, respectively where the AUROC and CI were 0.954, CI: 0.889-1.019; 0.979,CI: 0.892-1.004 and 0.989, CI: 1.00-1.001 respectively. D: is the ROC of the indices values corresponding to different histological stages of validation group. AUROC is 0.869, CI: 0.790-0.949

**Figure 6:**
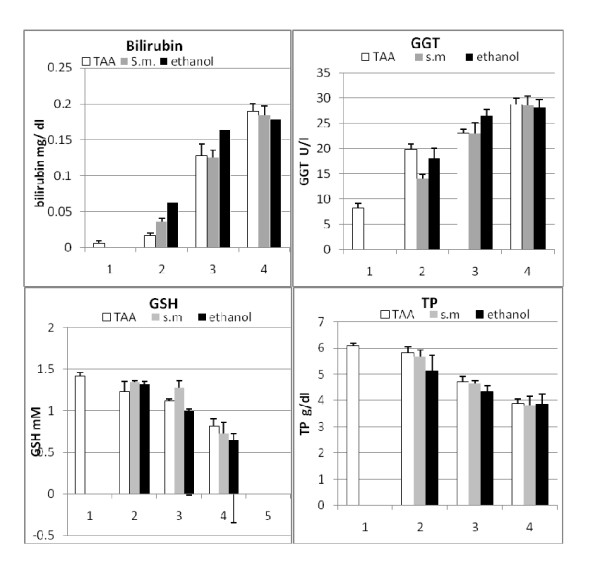
**Serum (non-invasive) markers of validation set measured at 4 time points**. The first time point (1) corresponds zero day of the normal untreated subset. For each mouse variables were combined in a logarithmic formula and used to scale the degree of fibrosis in validation group. Bil: bilirubin, γ-GT: gamma glutamyltranseferase, TP: Total protein and GSH: reduced glutathione

To validate the index, three groups of mice were treated with TAA, *s. mansoni*, or alcohol. Animals were sequentially sacrificed, where a subgroup of mice was killed every 10-15 days. The biochemical panel (bilirubin, γ-GT, T proteins and GSH) (Figure 5 and Figure 6) was evaluated and both the non- invasive score and the corresponding histological analysis were carried out independently. ROC curve was plotted. The area under ROC curve was 0.869 (95% Confidence Interval: 0.790-0.949) (Figure 4, panel D). Matching the obtained data revealed a high correlation between the non invasive score and both the stage and the grade, (0.947 and 0.859, respectively) (Table 7).

**Table 7:** Correlations and Kruskal-Wallis test between non-invasive score and the liver histology of both training and validation sets

		Histology			
		**Stage**		**Stage**	

	**Set**	**Spearman Correlation**	**Kruskal-Wallis**	**Spearman Correlation**	**Kruskal-Wallis**

**Non invasive Score**	**Training**	0.947 P < 0.01	χ^2 ^= 88.3 Sig 0.000	0.859 P < 0.05	χ^2 ^= 82.4 Sig: 0.000
					
	**Validation**	0.929 0.001	χ^2 ^= 76.4 Sig: 0.000	0.890 P < 0.001	χ^2 ^= 74.6 Sig: 0.000

## Discussion

Without diminishing the importance of histological assessment of liver fibrosis, non-invasive indices are moving towards accuracy and reliability. Previously proposed panels have included 2 to 7 markers. Increasing the number of variables may improve the accuracy, however it also increases the complexity and cost of the index. Previously reported indices included one (or more) variables from four categories. These categories include: (i) ECM proteins and enzymes involved in scar production, degradation, or hepatic stellate cells (HSC) activation, such as laminin, collagen, lysyl oxidase, prolyl hydroxylase, lysyl hydroxylase hyalauronic acid, or tissue inhibitors of metalloproteinases-1 (TIMP-1); (ii) Liver specific markers (bilirubin, transaminases or globulin); (iii) Haematological variables (platelets or PT); and (iv) Conjugated or derived lipids (apolipoprotein and cholesterol).

This work adds one more predictive non-invasive index that combines four simple, cheap and informative biochemical serum variables. The panel consists of an average number of variables (four), including three liver-specific, and one oxidative stress marker, simultaneously assessed in both normal (hypothetical standard) and tested samples. The aim was to formulate an index of liver fibrosis, which is experimentally induced with three different methods including: exposure to TAA, chronic alcohol intake and, for the first time, infection with *S. mansoni*, which predominantly exists in the Nile delta and upper-Egypt. Because the study was performed on mice, and due to the unavailability of animal model for HBV or HCV infection, viral hepatitis-induced liver fibrosis was not included. Also, the experiment was designed to non-invasively score the fibrotic changes during the compensated stages of fibrosis, where animals were maintained in the precirrhotic stages (< F4) to monitor the drug-mediated resolution of fibrosis.

Occupational TAA induced liver fibrosis in man is quite uncommon compared to alcohol, viral and *s. mansoni *related chronic liver diseases. It is, however a potent hepatotoxin that is rapidly eliminated and can cause cumulative injury [26]. In addition to the oxidative stress it generates, TAA decreases the level of some endogenous antioxidants such as α-tocopherol [27]. Consequently, it is anticipated that supplementation of mice with silymarin (a known anti-oxidant), will compensate the depletion of the vitamin and challenge the oxidant effect of TAA. Treatment of fibrosis in *S. mansoni*-infected mice, in contrast, required a specific eradication of the parasite with PZQ [28], followed by treatment with silymarin. This pattern of progressive and regressive fibrogenesis process was used to score the degree of scar formation (or resolution) using a dual approach of histological and non-invasive biochemical index.

The results revealed that oxidative stress commonly developed in mice with liver fibrosis as indicated by the depletion of serum GSH and elevated hepatic MDA. In such conditions, HSCs are activated and excessively produce ECM proteins (2-fold increase of hepatic collagen was observed). The over production of collagen was accompanied with changes in liver markers and the corresponding histological stage. We believe that the changes in the level (or the activity) of ECM proteins and enzymes, reported in some previous indices [29], follows the oxidative stress-mediated HSC activation. Novitskiy and his co-workers [30] found that treatment of HSC with ethanol led to the formation of ROS, activation of HSC and subsequently promotion of fibrogenesis. Thus, both GSH and hepatic MDA are able to monitor the initial steps rather than other ECM variables (collagens, HA, and other ECM proteins). Also, due to the complexity of ECM environment, inclusion of ECM variables may decrease the sensitivity of the index. Moreover, ECM proteins and enzymes are not exclusively liver-specific and may reflect impaired hepatic clearance [31]. Mizushima and co-workers [32], for instance, validated prolyl hydroxylase and tissue inhibitor of metalloproteinase (TIMP) as a marker of pancreatic fibrosis. Moreover, ECM molecules are largely affected by the balance between enzymes that control scar formation, stabilization and degradation. In addition certain pathological states, like pulmonary fibrosis, are associated with increased levels of certain fibrosis markers [33]. Previously we found that transglutaminase (liver derived enzyme known to stabilize the scar formation) did not show significant differences among patients with different grades of cirrhosis and HCC [34]. Also, the high correlations (*Spearman*) we obtained between GSH with both the histological stage and grade (-0.898 and -0.819, respectively) have nominated GSH as an integral member of the proposed index.

The remaining parameters included in the index are known to be directly involved in cellular pathogenesis and the synthetic capability of liver cells. ALT and AST have repeatedly used in hepatic fibrosis indices (individually, as a ratio or combined with other variables such as platelets [8,35,36]. ALT, in particular, was included in some non-invasive panels to indicate the necro-inflammatory activity [37]. Detection of fibrosis in HCV patients with normal transaminases [38] may decrease their validity as fibrosis markers. Bilirubin and albumin (the major fraction of plasma proteins) are traditionally among the biochemical parameters used to monitor the severity of liver disease. γ-GT is used as a marker of liver damage by alcohol intake [32]. This enhanced our predictive usefulness for the proposed index. The high correlations between liver histology (stage and grade) from one side and γ-GT (rather than ALT, AST, and ALP), bilirubin and T. Proteins from the other side (Table 4), have encouraged their combined involvement in the logarithmic formula. Liver dysfunction, leads to a decrease of the level of two of the selected markers (GSH and TP) and an increase the others (bilirubin and γ-GT). This predictive pattern was formulated by the mutual reversing the test/control ratio in the logarithmic formula.

The ground base is achieved in normal liver, where the ratio will be (or near to) "1". Accordingly, the index starts with "0", which corresponds to normal histological pattern. The highest scores (2, 1.5 and 1.8) matched the histological stages F3, F2 and F3 in groups II, V and X. A marked decrease in fibrosis index was observed in treated groups with index values ranging from 0.1 to 0.2.

Histologically, the highest fibrotic stage was observed in mice in groups II, V and X (Figure 2). Different treatments (with silymarin or PZQ), however, led to regression of the fibrotic stages to mild or moderate fibrosis in TAA groups or complete resolution of fibrosis in schistosomiasis and alcohol-induced fibrosis. The index was comparable to histological anaysis both following initial treatments with alcohol, TAA, and *s. mansoni *as well as following regression of fibrosis after treatment with silymarin and PZQ. High correlations were observed between the score and both grade and stage. Also, the values of AUROC inhances the potential usefulness of the index (Figure 4 and Tables 6). Within groups the index is correlated well with the corresponding histological stages, where in TAA, *S.mansoni *and alcoholism groups (*r *= 0.97, 0.84 and 0.97, respectively). Also, the data prospectively obtained from the validation set enhances the index reliability, where it matched the pathological stages and grades, which were independently assessed.

This index resulted in higher AUROC values and better identification of individual stages of fibrosis than the indices that are currently being used clinically in man. This could partly be because compared with these indices, it was studied in a more controlled and predictable environment over a shorter term. Nevertheless, its assessment in chronic liver disease in man may be warranted.

## Conclusion

This study assessed the fibrotic changes during the progression and regression of fibrosis. It provides a new experimental, reproducible and non invasive index of liver fibrosis, formulated in a logarthmic formula, which combined 3 common routine laboratory liver specific markers in addition to GSH. The index can predict the full range of precerrhotic stages and correlated well of the histological staging and grading. Different etiologies cause liver fibrosis in man including viral infection, *s mansoni*, ethanol, and autoimmune diseases. This index was utilized in mice experiments that assess the development and/or treatment of fibrosis. It could be used in future rodent models to assess damage and treatment for various hepatic toxins and pathogens. If such a model could be validated in prediction of fibrosis in man, it would minimize the need for liver biopsy to assess hepatic fibrosis.

## Competing interests

The data presented in this study is not influenced by our personal or financial relationship with other people or organizations. Also there are no financial or non financial competing interests that may cause embarrassment after the publication of the manuscript. 'The authors declare that they have no competing interests.

## Authors' contributions

**MH**: designed the study, generated and analyzed the data of the biochemical investigations performed in the study, put the mathematical formula used as an fibrosis index, prepared the manuscript for publication, supervised the animal care, treatments and he is the corresponding author. **IS**: carried out the infection of mice with schistosoma, performed the histological analysis and drafted the paragraphs concerned with the methodology of this method. **AB**: participated in the study design and coordination, participated in drafting the initial version of the manuscript. DB: Carried out the animals care, induced fibrosis in mice with TAA and ethanol, preformed animal treatment, sacrificing, sample collection, validated the proposed index in a separate experiment. **NS**: He critically revised the manuscript and largely participated in responding to the reviewer's concerns. All authors read and approved the final manuscript.

## Pre-publication history

The pre-publication history for this paper can be accessed here:

http://www.biomedcentral.com/1471-230X/10/53/prepub
